# Heterologous Caseins: The Role of Phosphorylation in Their Functionality and How to Achieve It

**DOI:** 10.3390/biom15071031

**Published:** 2025-07-17

**Authors:** Soledad Mora Vásquez, Santiago García-Jacobo, Guy A. Cardineau, Silverio García-Lara

**Affiliations:** 1 Tecnologico de Monterrey, School of Engineering and Sciences, Ave. Eugenio Garza Sada 2501 Sur, Col: Tecnológico, Monterrey 64700, NL, Mexicosgarcialara@tec.mx (S.G.-L.); 2 Beus Center for Law and Society, Arizona State University, Mail Code 9520, 111 E. Taylor Street, Phoenix, AZ 85004-4467, USA

**Keywords:** phosphoprotein, post-translational modification, milk protein engineering, recombinant casein, kinase

## Abstract

Heterologous expression of caseins in non-mammalian systems offers a sustainable and scalable alternative for producing milk proteins, with potential applications in the food and biopharmaceutical industries. However, a significant challenge in these systems is achieving proper phosphorylation, a critical post-translational modification required for casein functionality and stability. This review explores the current state of research on heterologous casein production, with a particular focus on the biological and technical hurdles associated with phosphorylation. Specifically, we examine the absence of the mammalian-specific kinase Fam20C in plant and yeast systems and the broader lack of secretory kinase machinery in bacteria, which collectively contribute to impaired phosphorylation fidelity. While some endogenous kinases may partially compensate, they are typically insufficient to replicate the phosphorylation pattern required for functionality. We evaluate potential strategies to address these limitations, analyze the role of phosphorylation in casein functionality, provide insights into existing patents and experimental approaches, and highlight ongoing research efforts. By synthesizing current knowledge and proposing new avenues for innovation, this review aims to provide a roadmap for the successful production of functional heterologous caseins.

## 1. Introduction

Caseins, a group of essential phosphoproteins, play a crucial role in the nutritional and functional properties of mammalian milk. Four major types—alpha-S1, alpha-S2, beta, and kappa caseins (αs1, αs2, β, and κ)—have been identified, with their proportions varying significantly between species. For instance, αs1-casein is abundant in cow’s milk, whereas it is present in only trace amounts in human milk [1].

**Figure 1:**
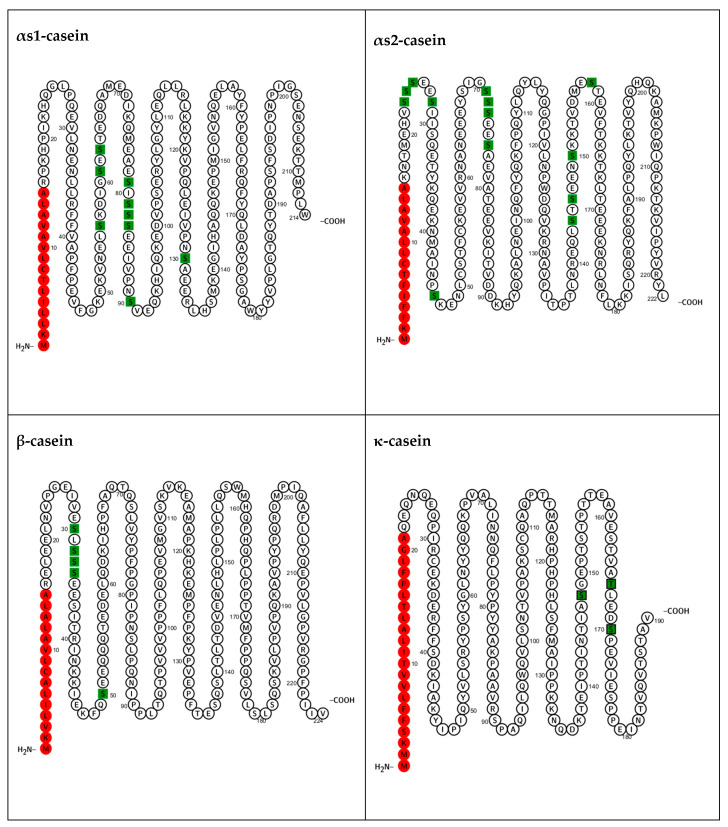
Phosphorylation sites (in green) and signal peptides (in red) in bovine casein sequences. Figure made using Protter (version 1.0) [4].

In bovine milk, caseins primarily undergo phosphorylation at specific serine residues; however, in the case of κ-casein, phosphorylation can also occur at a threonine residue [2] . The αs1-, αs2-, and β-caseins generally possess approximately 8, 10–13, and 5 phosphate groups, respectively. Nonetheless, these values represent common averages and do not reflect the full range of naturally occurring phospho-variants. For instance, αs1-casein exists in at least two major phosphorylated forms, αs1-CN-8P and αs1-CN-9P, which have been shown to be genetically distinct traits influenced by different gene loci and regulatory mechanisms [3]. As illustrated in Figure 1, phosphate groups in αs1-casein are predominantly located in the central region, while in αs2-casein, they are distributed across both central and N-terminal regions. β-casein’s phosphate groups are concentrated at the N-terminal. κ-casein typically has only 1–3 phosphorylated residues located near its C-terminal end. For detailed sequence information, see the UniProt protein accessions: P02662 (αs1-casein), P02663 (αs2-casein), P02666 (β-casein), and P02668 (κ-casein).

All bovine caseins contain phosphorylated residues, though the extent of phosphorylation varies between casein genetic variants and isoforms, with the mechanisms governing this variability still not fully understood [5,6].

Heterologous synthesis of caseins has gained considerable attention as a viable alternative to conventional dairy production, offering potential solutions to both ethical and environmental challenges associated with traditional methods [7]. Given the potential use of recombinant caseins in infant formulas, functional foods, and therapeutic applications, it is important to consider the genetic polymorphisms of caseins and their physiological relevance. Casein polymorphisms, which involve variations in amino acid sequences and post-translational modifications, can influence digestion, bioactive peptide release, and allergenicity. As a notable example, the A1 variant of β-casein can yield the bioactive peptide β-casomorphin-7 (BCM-7) during digestion, which has been linked to various health conditions, while some studies suggest it may also have protective gastrointestinal effects [8].

Heterologous caseins are produced through genetic engineering in non-mammalian organisms such as bacteria, yeast, and plants. One unresolved question is whether heterologous caseins can be correctly phosphorylated in these new hosts. The main objective of this review is to examine prior research on heterologous casein expression to determine whether the phosphorylation patterns match those expected. To this end, a comprehensive search was performed using both the scientific literature and patent databases. Literature searches were conducted on electronic databases such as PubMed, Web of Science, SciELO, and ScienceDirect. To supplement this, patent databases, including Google Patents, USPTO, Espacenet, and WIPO, were also utilized to identify patents related to recombinant and heterologous casein expression. The search terms included “recombinant casein,” “heterologous casein,” and “casein expression in bacteria, yeast, or plants.” The search targeted articles and patents published in English that focused on the expression of caseins. No specific time frame was applied, allowing for the inclusion of all relevant publications to date. The inclusion criteria encompassed studies and patents that provided detailed insights into the expression systems used, including bacterial, yeast, or plant platforms, and their efficacy in producing functional caseins.

## 2. In Vivo Casein Phosphorylation

Phosphorylation is a well-established post-translational modification (PTM) in eukaryotic cells, broadly contributing to the regulation of essential cellular processes. These processes encompass signal transduction, protein–protein interactions, cell growth, differentiation, the stabilization of three-dimensional protein structures, subcellular localization, and protein turnover, among others [9,10]. This process involves the covalent attachment of a phosphate group to serine, threonine, or tyrosine residues, catalyzed by kinases, and can be reversed by phosphatases.

In 1969, two enzymes, originally designated as protein kinase I (CKI) and protein kinase II (CKII), were mistakenly identified as “casein kinases” due to their capacity to phosphorylate casein in vitro. However, it has since been demonstrated that these enzymes phosphorylate a broad range of substrates. To avoid further misidentification and distinguish them from the authentic casein kinase, their nomenclature was revised to protein kinases [11,12,13]. The enzyme responsible for authentic casein kinase activity was initially identified in 1977 when it was isolated from bovine mammary glands, where its capacity to phosphorylate casein was confirmed. Despite extensive biochemical investigations, its precise identity remained elusive until 2012, when it was recognized as Fam20C (Family with Sequence Similarity 20). Fam20C is localized within the Golgi apparatus and is secreted along with its substrates, distinguishing it from CKI and CKII, which are primarily localized in the nucleus and cytoplasm. Although intracellular phosphorylation is well-studied, the roles and regulation of protein phosphorylation within the secretory pathway remain largely unclear [14]. Fam20C may phosphorylate proteins either prior to secretion or following exocytosis, thus contributing to extracellular protein phosphorylation [15]. Fam20C is the primary kinase responsible for phosphorylating not only casein but also the majority of secreted proteins. Fam20 kinases are highly conserved across the animal kingdom, from sponges to mammals [16].

## 3. Why Are Phosphate Groups Important?

As outlined in the following sections, phosphate groups in casein molecules are essential for both physiological functions and industrial applications.

### 3.1. Calcium Binding and Bioavailability

The biological role of caseins—particularly in the formation of casein micelles—is intrinsically linked to their ability to bind and transport calcium to the neonate, a function that relies on the presence of phosphate groups, especially in highly phosphorylated proteins like β-casein. These phosphate groups facilitate the interaction with calcium ions, enabling the formation of micellar structures that stabilize calcium and phosphate in milk. This stabilization is essential for ensuring the bioavailability of these minerals for bone and dental development in mammals. Moreover, proper phosphorylation prevents pathological calcification in the mammary gland. In the absence of sufficient phosphorylation, caseins lose their capacity to form micelles, thereby compromising the efficient and safe delivery of calcium in a bioavailable form [17].

### 3.2. Milk Stability and Nutritional Quality

Phosphate groups in caseins are key to the structural integrity and nutritional functionality of milk. By facilitating the formation of casein micelles, these groups prevent the precipitation of calcium and phosphate, thereby preserving milk in a stable, liquid form. This micellar stability supports a slow and sustained release of nutrients, which is particularly important for the growth and development of young mammals. Furthermore, phosphate groups influence the physical properties of the micelles and their digestive behavior, contributing to clot firmness in the stomach. This affects how fat is entrapped and how nutrients are released over time, with higher phosphate levels leading to firmer clots and extended gastric retention. Such properties are especially beneficial in species with infrequent nursing patterns. Additionally, micelles prevent calcium deposits in mammary tissue, protecting the gland from pathological calcification and ensuring milk remains both safe and nutritious [18]. Beyond these roles, phosphate groups significantly contribute to the chaperone activity of caseins by enhancing structural flexibility and solubility under thermal or reductive stress. This function helps stabilize partially unfolded dietary or endogenous proteins, preventing their aggregation and preserving their bioavailability. Such molecular adaptability, driven by phosphoserine residues, not only mimics the behavior of small heat shock proteins but also reinforces the idea that milk is not only a source of essential nutrients but also a protective matrix that enhances their stability and functionality in complex physiological environments [19,20,21,22,23].

### 3.3. Dairy Industry Coagulation Processes

Phosphorylation is equally important in the dairy industry, particularly for coagulation processes. Calcium-sensitive caseins (αs1, αs2, and β) rely on their phosphate groups to interact with calcium ions, which is essential for coagulation during cheese production and other dairy formulations [24]. Without proper phosphorylation, these caseins would not interact efficiently with calcium, resulting in poor curd formation and subsequently affecting the product yield and quality [25].

### 3.4. Biomedical Applications

Beyond food applications, phosphorylated caseins have gained attention in biomedical fields, including drug delivery systems and tissue engineering, due to their biocompatibility and functional versatility. By leveraging casein’s structural flexibility, molecular chaperone capabilities, and interaction with minerals, new nanosystems can be developed for controlled and sustained drug release. Additionally, the functional attributes of casein-based nanosystems, such as their capacity to respond to specific environmental conditions (e.g., pH and temperature), allow for precision in therapeutic applications, including the treatment of iron deficiency anemia and neurodegenerative diseases [26,27]. As researchers continue to explore these cutting-edge applications, phosphorylation groups in casein are integral to optimizing its efficacy in emerging biomedical technologies.

### 3.5. Bioactive Peptides in Digestion

Phosphorylated caseins serve as precursors to bioactive phosphopeptides released during digestion. These phosphopeptides exhibit diverse physiological activities, including antimicrobial, antihypertensive, and immunomodulatory effects. Phosphorylation plays a crucial role in enabling these peptides to bind minerals, resist premature degradation, and be effectively released during gastrointestinal digestion, thereby contributing to the health benefits associated with milk and dairy product consumption [28,29]. Casein-derived phosphopeptides, also known as casein phosphopeptides (CPPs), are particularly noted for their ability to enhance the absorption of minerals such as calcium, iron, and zinc, owing to their phosphate residues and strong chelating capacity. This mineral-binding ability varies depending on whether CPPs are released from αs1-, αs2-, or β-casein subunits, and their physicochemical properties—including stability at acidic pH and high solubility—make them attractive for nutritional delivery applications. By chelating essential minerals, CPPs contribute to improved bioavailability and further emphasize the nutritional significance of phosphorylated caseins in the diet [30,31].

### 3.6. Hypoallergenic Properties

Phosphorylation also affects the allergenic properties of caseins. The absence of phosphate groups has been linked to allergic reactions in certain individuals [32,33]. This suggests that the proper phosphorylation of caseins can contribute to hypoallergenic properties, which are significant for the development of specialized dairy products.

**Figure 2:**
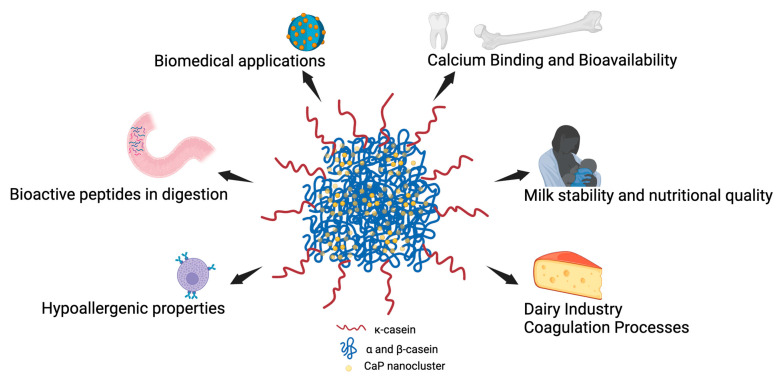
Role of Phosphate Groups in Caseins. The central image displays a casein micelle structure, with phosphate groups (indicated by calcium phosphate nanoclusters). Created in BioRender. Mora vasquez, S. (2025) https://BioRender.com/l13l221 (accessed on 24 June 2025).

Figure 2 illustrates the critical role of casein phosphorylation in both dairy science and biomedical applications. Phosphorylated caseins enable calcium binding, forming stable micelles essential for bioavailable calcium delivery, milk stability, and coagulation processes in cheese production. Proper phosphorylation also contributes to hypoallergenic properties in dairy products and the formation of bioactive peptides during digestion, which have health-promoting effects. In biomedical fields, phosphorylated caseins are utilized in nanosystems for targeted drug delivery, leveraging their structural flexibility and mineral-binding capabilities for therapeutic applications.

Phosphorylation plays a vital role in both emerging biomedical technologies and dairy product formation. In casein micelles, phosphorylation contributes to calcium phosphate bridging and structural integrity, while κ-casein’s post-translational modifications, including glycosylation and phosphorylation, are essential for stabilizing the micelle surface and preventing aggregation. Notably, β-casein, and to some extent αs-caseins, can also self-assemble into micelle-like structures under appropriate ionic and thermal conditions, although these assemblies differ in stability from native micelles and are influenced by their phosphorylation status [34,35,36,37]. Comprehensive reviews detailing casein micelle structure and assembly models are available for readers seeking deeper insight into these aspects [25,27,34,38,39]. Therefore, ensuring appropriate post-translational modifications of heterologous caseins is necessary to engineer artificial micelles that accurately replicate the stability, solubility, and functional properties of natural micelles.

## 4. Heterologous Caseins

### 4.1. Caseins Expressed in Bacteria

Since the early 1980s, caseins from various species, including mouse and bovine, have been cloned and characterized using recombinant DNA techniques, as shown in Table 1. The initial research focused on isolating and cloning cDNAs encoding these caseins, followed by their expression in *Escherichia coli*. These studies enabled the production of recombinant caseins for further exploration of their structural and functional properties. Subsequent research employed site-directed mutagenesis to systematically alter casein sequences, offering deeper insights into their molecular behavior. These advancements enhanced our understanding of casein functionality and also paved the way for applications in genetic engineering and dairy biotechnology.

**Table 1:** Chronological overview of studies on heterologous casein expression in bacteria.

Casein	Host Organism	Purification/Detection Methods	Phosphorylation Analysis	Result	Level of Expression	Ref.
Rat C2 casein (αs2-casein)	*E. coli*	Protein extraction, radiolabeling, and radioimmunoassay	-	Undetermined	-	[40]
Bovine β-casein	*E. coli*	Protein extraction, SDS-PAGE, and Western blot (WB)	-	Undetermined	-	[41]
Bovine αs1-casein	*E. coli*	Pulse-chase labeling, SDS-PAGE, and WB	Phosphatase treatment and SDS-PAGE	Not phosphorylated		[42]
Bovine β-casein	*E. coli*	SDS-PAGE, WB, and immunochemical staining	-	Undetermined		[43]
Bovine β-casein	*E. coli*	Isoelectric precipitation, RP-HPLC, SDS-PAGE, and Western blotting.	-	Undetermined		[44]
Human β-casein	*E. coli*	Ion exchange chromatography, RP-HPLC, SDS-PAGE, and Western blotting	SDS-PAGE stained with ethyl stains—all mass spectrometry analysis	Not phosphorylated		[45]
Bovine β-casein	*E. coli*	FPLC, SDS-PAGE, and WB	Urea-PAGE, SDS-PAGE, negative-ion LC–ESI–MS	Phosphorylated	500 mg/L	[46]
Human αs1-casein	*E. coli*	Ni-NTA affinity purification and protein sequencing	-	Undetermined	25% of the total cell protein	[47]
Bovine αs1-casein	*E. coli*	SDS-PAGE	-	Undetermined	18.00%	[48]
Human β-casein	*E. coli*	Ion exchange chromatography and SDS-PAGE		Not phosphorylated		[49]
Bovine β-casein	*E. coli*	SDS-PAGE	-	Not phosphorylated	-	[50]
Bovine αs1-casein	*E. coli*	Ni-NTA affinity purification, dialysis, protein quantification, and SDS-PAGE	-	Undetermined	1.6 mg/L	[51]
Bovine β-casein	*E. coli*	Isoelectric precipitation, preparative reverse-phase HPLC, and SDS-PAGE.	Infusion mass spectrometry of purified phosphopeptides	Partially phosphorylated	200 mg/L	[52]
Bovine αs1-casein	*E. coli*	Ni-NTA affinity purification, SDS-PAGE, and Western blotting	-	Undetermined	60 mg/L	[53]
Bovine αs1-casein	*Lactococcus lactis*	Ni-NTA affinity purification, SDS-PAGE, and Western blotting	-	Undetermined	-	[54]
Bovine β-casein	*E. coli*	Ni-NTA affinity purification, SDS-PAGE, and Western blotting	-	Undetermined	-	[55]
Bovine αs1-casein and human αs1-casein	*E. coli*	SDS-PAGE	-	Undetermined	1.45 g/L	[56]

The most commonly used bacterial system for expressing caseins is *Escherichia coli*, due to several key advantages. Bacterial cultures are relatively inexpensive to maintain and exhibit rapid growth, making them highly cost-effective for large-scale production [57]. Furthermore, the genetic manipulation of bacterial systems is straightforward, allowing for the introduction of mutations or structural modifications in caseins. This feature is particularly valuable for studying structure–function relationships or engineering caseins with specific properties, such as enhanced resistance to proteolysis. Moreover, bacterial systems offer scalability, which is advantageous in industrial applications where large-scale production of caseins is required for biotechnology and food science purposes. In our review, yields ranging from 1.6 mg/L in small-scale setups to 1.45 g/L in larger-scale systems were observed, demonstrating the versatility of *E. coli* in accommodating various production needs. Alternatives to *E. coli* for the heterologous expression of casein bioactive peptides include *Lactococcus lactis* and *Streptococcus thermophilus*, which are both commonly used in the dairy industry [58]. These lactic acid bacteria are well-established in the fermentation of dairy products such as processed cheeses, yogurt, and functional foods. Their ability to produce biofunctional molecules that contribute to consumer health makes them attractive hosts for recombinant protein expression, particularly in applications related to food science and biotechnology [54].

While phosphorylation is now recognized as a significant regulatory mechanism in bacterial metabolism [59], wild-type bacterial systems lack the secretory kinase machinery required to reproduce the specific phosphorylation patterns essential for functional caseins, such as those mediated by mammalian Fam20C.

In an effort to study casein phosphorylation in bacterial systems, Thurmond et al. co-expressed human β-casein with protein kinase CK2 in *Escherichia coli* using a polycistronic construct that encoded the casein protein along with both the α- and β-subunits of human CK2 [46]. This method successfully mimicked phosphorylation, achieving high levels of phosphorylated recombinant human β-casein, and facilitated a deeper understanding of its effects on micelle formation and bioactivity.

While co-expression of human β-casein with protein kinase CK2 successfully replicated the physiological phosphorylation pattern, this approach was less effective for bovine β-casein. In experiments, bovine β-casein co-expressed with CK2 achieved a much lower phosphorylation level compared to the fully phosphorylated 5P state observed in the naturally produced protein from the mammary gland. This reduced phosphorylation suggests that CK2 does not efficiently recognize the phosphorylation sites in bovine β-casein, likely due to differences in its primary sequence or structural conformation. In contrast, human β-casein demonstrated better substrate recognition by CK2 [52]. CK2 phosphorylates serine residues within both canonical and some non-canonical motifs, but phosphorylation efficiency and occupancy are site-dependent. In human β-casein, the serine clusters align well with CK2 consensus sites, facilitating near-complete phosphorylation. In bovine β-casein, although clusters of serine residues exist, only some are in canonical CK2 recognition sites, and even within these clusters, not all potential sites are phosphorylated simultaneously [52,60]. These findings imply that co-expression with CK2 may not be the most suitable approach for fully phosphorylating bovine caseins without sequence modifications to improve kinase recognition.

Phosphorylation plays a key role in calcium binding and micelle formation, and this modification is poorly replicated in bacterial systems, even with co-expression strategies such as those involving protein kinase CKII. The result is often incomplete phosphorylation or protein misfolding, leading to functionally compromised caseins. The absence of post-translational modifications, coupled with the potential introduction of bacterial endotoxins, also presents regulatory challenges when recombinant caseins are intended for therapeutic use or human consumption [61].

In many of the studies summarized in Table 1, phosphorylation status is indicated as “undetermined,” reflecting the absence of any reported analysis. Caseins expressed in bacterial systems have often been used for applications such as structural studies, peptide mapping, or enhancing nutritional content, where replicating native post-translational modifications was not a central objective. As a result, phosphorylation—though critical to native casein functionality—was frequently excluded from the experimental scope. Additionally, given the known absence of secretory kinases in bacterial systems, researchers may have assumed minimal or no phosphorylation, reducing the perceived necessity for its analysis. This, combined with the technical challenges of phosphorylation detection, contributed to its omission from many of the referenced studies.

### 4.2. Caseins Expressed in Yeast

Yeast expression systems, such as *Saccharomyces cerevisiae* and *Pichia pastoris*, provide a versatile platform for producing bovine caseins due to their ability to perform post-translational modifications like glycosylation and phosphorylation. These features make yeast systems invaluable for exploring casein’s structural and functional properties. Expression levels ranging from 0.6 mg/L to 1 g/L, as shown in Table 2, demonstrate their suitability for both research and industrial applications.

**Table 2:** Overview of studies on heterologous casein expression in yeast.

Casein	Host Organism	Purification/ Detection Methods	Phosphorylation Analysis	Result	Level of Expression	Ref.
Bovine β-casein	*Saccharomyces cerevisiae*	Ion exchange chromatography, SDS-PAGE, and WB	Phosphatase treatment and Urea-PAGE	Yeast casein and bovine casein displayed the same mobilities in urea gels before and after dephosphorylation	-	[62]
Bovine β-casein	*Saccharomyces cerevisiae*	SDS PAGE and WB	-	Undetermined	10 mg/L	[63]
Bovine β-casein	*Pichia pastoris*	Ammonium sulfate precipitation, chromatography, SDS-PAGE, and WB	.	Undetermined	1 g/L	[64]
Human αs1-casein	*Saccharomyces cerevisiae*	SDS-PAGE and Western blot (WB)	-	Undetermined	0.6 mg/L	[65]
Bovine β-casein (mutant)	*Pichia pastoris*	Fractionation using ammonium sulfate precipitation, Concanavalin A-Sepharose affinity chromatography, anion-exchange HPLC, SDS-PAGE, and WB	Phosphatase treatment and Urea-PAGE	Phosphorylated and glycosylated	-	[66]

Yeast systems appear to have the capability to phosphorylate caseins in vivo, suggesting their potential to mimic some post-translational modifications observed in mammalian systems. However, the extent and site-specific phosphorylation levels of yeast-expressed caseins remain underexplored, as many studies have prioritized secretion efficiency, yield optimization, and glycosylation analysis over detailed phosphorylation profiling. Given that phosphorylation analysis requires specialized proteomic approaches, the phosphorylation status for several yeast-expressed caseins remains “undetermined” in Table 2.

While yeast systems can also introduce O-glycosylation during casein expression, which may affect the protein’s structural and functional properties, it is critical to note that such modifications do not reflect the native post-translational landscape of animal-derived caseins. This divergence could impact key functionalities, including calcium-binding capacity and micelle formation, which are essential for the nutritional and structural roles of caseins. Nevertheless, this glycosylation has been observed primarily in certain *S. cerevisiae* strains, indicating variability across yeast systems [62].

A notable study by Choi and Jimenez-Flores leveraged the eukaryotic machinery of yeast to explore targeted modifications in bovine β-casein, introducing N-linked glycosylation sites while preserving the phosphoseryl cluster, thereby maintaining potential phosphorylation while exploring the effects of additional modifications [64].

Overall, yeast expression systems offer several advantages for recombinant casein production, including the ability to perform eukaryotic-like phosphorylation, ease of genetic manipulation, and scalability. However, the variability in phosphorylation efficiency and the potential introduction of non-native glycosylation highlight the need for further systematic studies focused explicitly on phosphorylation profiling to assess the suitability of yeast-derived caseins for applications requiring structural fidelity and functional equivalence to native proteins. 

### 4.3. Caseins Expressed in Plants

Plants are increasingly recognized as viable platforms for producing recombinant proteins, including caseins, due to advantages such as low production costs, scalability through agricultural operations, and biosafety benefits [67]. While plants cannot fully replicate mammalian post-translational modifications, they are capable of some that may be adequate for specific applications. However, phosphorylation, a key post-translational modification for caseins, remains a significant challenge in plant-based systems.

Table 3 highlights studies on heterologous casein expression in plants, revealing that only two research articles have been published to date. Despite this limited academic research, there is a significant body of patents exploring the use of plants as platforms for recombinant protein production, indicating considerable interest in their industrial applications.

**Table 3:** Studies on heterologous casein expression in plants.

Casein	*Host organism*	Purification/ Detection Methods	Phosphorylation Analysis	Result	Level of Expression	Ref.
Human β-casein	*Solanum tuberosum*	SDS-PAGE and WB	-	The apparent molecular weight was slightly smaller than that of phosphorylated casein	0.01%	[68]
Bovine β-casein	*Glycine max*	SDS-PAGE and WB	Mass spectrometry (MALDI-MS)	Not phosphorylated	0.1–0.4%	[69,70]

The expression of caseins in plant-based systems, such as potato and soybean, has demonstrated potential for recombinant milk protein production. However, a persistent challenge is the absence of phosphorylation patterns observed in mammalian caseins. Unlike mammals, plants do not express the FAM20C kinase, which is critical for the proper phosphorylation of caseins. While plants possess endogenous phosphorylation mechanisms for their own proteins, they have not successfully phosphorylated recombinant caseins. This limitation may arise from the lack of precise temporal and spatial regulatory conditions required for accurate phosphorylation, raising concerns regarding functionality and safety.

Table 4 presents a selection of patents in this field, highlighting the increasing industrial interest in utilizing plant systems for recombinant milk protein production. These patents encompass various applications, including the development of animal-free dairy alternatives, such as αS1 and αS2 caseins, as well as modified caseins engineered for tailored functionalities. Additionally, some patents focus on novel food products that integrate milk and non-animal proteins, reflecting efforts to address the growing demand for sustainable and scalable dairy alternatives.

**Table 4:** Recent patents on heterologous casein expression in biological systems.

Patent Number	Title	Assignee	Filing Date	Expression System	Key Innovations/Application	Ref.
US20240251810A1	Increased yield of milk protein per acre	Nobell Foods Inc.	2024-03-07	Plants, yeast, and bacteria	Increased production of recombinant milk proteins	[71]
US20240376167A1	Animal free alpha s1 and alpha s2 casein-fusion milk protein, process for preparing the same	Heni Innovation Private Ltd.	2023-06-21	Plants, yeast, and bacteria	Vectors and expression cassettes for the expression of animal-free milk proteins in host cells.	[72]
US20240360190A1	Recombinant vectors for expression of animal free milk protein and process for preparing the animal free milk protein	Heni Innovation Private Ltd.	2024-04-17	Plants, yeast, and bacteria	Vectors and expression cassettes for the expression of animal-free milk proteins in host cells.	[73]
WO2024194831A1	Expression of animal myofibrillar proteins in plants	MOOLEC SCIENCE LIMITED	2024-03-21	Plants	Higher expression yields by using superior hosts	[74]
US12077798B2	Food compositions comprising recombinant milk proteins	Nobell Foods Inc.	2023-12-13	Plants	Transgenic plants stably expressing recombinant fusion milk proteins at ≥1% of total soluble protein for sustainable production.	[75]
US12139737B2	Host cells comprising a recombinant casein protein and a recombinant kinase protein	Nobell Foods Inc.	2023-09-30	Plants, yeast, and bacteria	Casein phosphorylation	[76]
WO2024013749A1	Methods for producing functional milk proteins in a plant cell, products, and uses thereof	IMAGENE FOODS	2023-07-13	Plants	Biosynthesis of functional milk proteins	[77]
WO2024015365A1	Recombinantly expressed proteins in chemoautotrophic microorganisms for use as food ingredients	KIVERDI, INC.	2023-07-11	Plants, algae, yeast, and bacteria	Proteins that are recombinantly expressed in chemoautotrophic microorganisms.	[78]
WO2023197002A2	Modified casein proteins	MOZZA FOODS, INC.	2023-04-07	Plants	Production of differentially glycosylated recombinant casein proteins	[79]
US20230146346A1	Recombinant fusion proteins for producing milk proteins in plants	Nobell Foods Inc.	2023-01-20	Plants	Production of recombinant fusions proteins and food compositions comprising the same.	[80]
WO2023141256A2	Methods for production of animal-free honey and milk substitutes	Sifotek, AS	2023-01-20	Bacteria, yeasts, or fungi	Production of milk proteins through precision fermentation.	[81]
WO2023092005A1	Compositions, methods and systems for phosphorylation of proteins in plants	MOZZA FOODS, INC.	2022-11-17	Plants	Phosphorylation of proteins in plants	[82]

In the patent landscape, while plant-based expression systems—as well as alternative hosts such as bacteria, yeast, and fungi—hold promise for recombinant casein production, many patents lack comprehensive analyses of phosphorylation patterns and potential allergenicity. Addressing these gaps is crucial to fully realizing the potential of plant-based platforms for functional and sustainable dairy innovations.

A notable patent (US12139737B2) outlines a strategy for achieving phosphorylation of recombinant caseins in plants through co-expression with kinases [76]. The invention involves expressing caseins along with enzymes capable of post-translational modifications, such as kinases or glycosyltransferases. Of particular significance is the incorporation of Fam20C, a kinase that phosphorylates Ser-X-Glu/pSer motifs, thereby enhancing casein phosphorylation. This approach represents a promising strategy for replicating mammalian phosphorylation patterns in plant-based expression systems, with potential applications in biotechnology and nutrition.

Similarly, patents WO2024013749A1 and WO2023092005A1 describe the genetic modification of plant cells to express mammalian caseins (e.g., αS1-casein, αS2-casein, β-casein, and κ-casein) in conjunction with at least one kinase promoting phosphorylation [77,82]. Notably, these patents highlight that the recombinant plant-derived caseins are coagulable and capable of forming functional micelles, essential for their structural and functional integrity in food applications.

Collectively, these patents represent a paradigm shift toward plant-based expression systems as viable platforms for animal-free dairy production, offering new opportunities for scalability, functionality, and sustainability in the food industry. These advancements highlight the growing interest in plant-derived dairy alternatives, aiming to reconcile sustainability imperatives with the biochemical properties of mammalian caseins.

## 5. Phosphorylation Analysis of Heterologous Casein

Recombinant caseins can be purified using ion-exchange chromatography; however, detection and purification are challenging when the casein lacks a tag or fusion protein, such as a reporter gene, to facilitate isolation. This complexity arises due to the heterogeneous nature of the expressed proteins and their propensity to form stable associations or aggregates with other proteins during fractionation processes. Furthermore, heterogeneity observed during ion exchange chromatography may be attributed to variations in the number of phosphate groups associated with the protein. A range of complementary analytical techniques is required to characterize phosphorylation patterns accurately.

### 5.1. Mass-Spectrometric Approaches

Mass spectrometry (MALDI-MS) has been employed for analyzing casein phosphorylation. Matrix-assisted laser desorption/ionization mass spectrometry (MALDI-MS) allows for the detection of phosphorylated peptides after enzymatic digestion of the purified casein. In the workflow described by Philip et al. (2001) [70], casein was first enriched from transgenic seed extracts using affinity chromatography with anti-casein antibody coupled to CNBr-activated Sepharose 4B, followed by further separation by two-dimensional SDS-PAGE prior to digestion and MALDI-MS analysis. This technique is highly sensitive and can provide detailed information on the exact sites of phosphorylation [70]. Another commonly used method is negative-ion liquid chromatography–electrospray ionization–mass spectrometry (LC–ESI–MS), which helps in identifying the phosphorylated peptides and quantifying their phosphate content [46]. Mass spectrometry is a highly sensitive analytical method that detects specific ions and yields detailed information about the molecular weight and structural properties of proteins. Additionally, MALDI TOF/TOF and negative-ion electrospray ionization tandem mass spectrometry (ESI-MS/MS) have been employed for phosphoprotein and phosphopeptide analysis, providing enhanced fragmentation and quantitative capabilities for site-specific phosphorylation mapping [83]. Research has also demonstrated that phosphopeptide enrichment using IMAC or TiO_2_ prior to mass spectrometry analysis can significantly improve the detection and characterization of phosphorylation sites [84,85].

### 5.2. Conventional SDS-PAGE

SDS-PAGE (Sodium Dodecyl Sulfate-Polyacrylamide Gel Electrophoresis) is another technique used to analyze phosphorylation. Phosphorylated caseins may be visualized by staining the gels with dyes like Ethyl Stains-All, which selectively binds to phosphorylated proteins, making them visible in the gel [45]. SDS-PAGE can also serve as a useful tool to provide an indication of whether a casein is phosphorylated by comparing its molecular weight to that of a known standard. For example, plant-produced β-casein was observed to migrate as a single band with a molecular weight of approximately 30 kDa, which was 1–1.5 kDa smaller than the phosphorylated control [68]. However, this method only offers an approximation and cannot provide absolute certainty about phosphorylation status, as additional confirmation through more specific analytical techniques is required.

### 5.3. Urea-PAGE Coupled to Phosphatase Assays

Urea-PAGE can separate phosphorylated proteins based on changes in their charge and molecular weight due to phosphorylation, making it useful for visualizing shifts in protein migration. This technique can also be combined with Western blotting using specific antibodies, such as those targeting casein, which enhances the detection and specificity of phosphorylated proteins. This combination allows for a more targeted analysis of phosphorylation events, providing additional confirmation of results. Phosphatase treatment and Urea-PAGE are used to confirm the presence and number of phosphate groups on caseins. Treating the casein with phosphatases, such as alkaline phosphatase or tyrosine phosphatase, can remove phosphate groups, allowing comparison between treated and untreated samples. The phosphorylation status is then evaluated using urea-polyacrylamide gel electrophoresis (Urea-PAGE), which separates proteins based on their molecular weight and charge. Urea-PAGE is particularly useful for detecting changes in protein charge due to phosphorylation [66].

Using Urea-PAGE instead of mass spectrometry to detect phosphorylation offers both advantages and disadvantages. One advantage of Urea-PAGE is its simplicity and accessibility, as it requires less specialized equipment and is generally more cost-effective compared to mass spectrometry.

However, Urea-PAGE has several limitations compared to mass spectrometry. It is less sensitive, meaning it may not detect low-abundance phosphorylated proteins or subtle phosphorylation events. Urea-PAGE also lacks the ability to provide detailed information about the specific phosphorylation sites or the degree of phosphorylation on a given protein. In contrast, mass spectrometry offers high precision, allowing for the identification of exact phosphorylation sites and the quantification of phosphorylation levels. Additionally, Urea-PAGE may struggle to resolve proteins with similar molecular weights or charge characteristics, potentially leading to overlapping signals that complicate interpretation. While the combination of Urea-PAGE with Western blotting can improve detection, it still cannot match the comprehensive depth of analysis that mass spectrometry provides.

### 5.4. Phos-Tag™-Based Strategies

Phos-tag™ acrylamide electrophoresis is now widely regarded as a rapid, high-resolution method for distinguishing phosphorylated from non-phosphorylated protein isoforms. Phos-tag gel electrophoresis enables the separation of protein isoforms based on their phosphorylation patterns by incorporating a phosphate-binding metal complex within the polyacrylamide gel matrix, which retards the migration of phosphorylated proteins relative to their non-phosphorylated counterparts. [86,87]. When β-casein is analyzed using this method, multiple discrete bands corresponding to different phospho-site configurations are observed, while dephosphorylated β-casein appears as a single band. Prior to Western blotting, gels require EDTA treatment to remove metal ions and release phosphorylated proteins from the Phos-tag complex, ensuring effective transfer. The technique thus provides a high-resolution, semi-quantitative means to assess the phosphorylation status of casein and other phosphoproteins [87].

Immobilized Phos-tag ligands on agarose or magnetic beads extend the utility of the chemistry to selective enrichment. Under mildly alkaline conditions, phosphorylated proteins are captured with high affinity, while non-phosphorylated species are efficiently washed away. Elution—typically achieved by competitive phosphate or metal chelation—produces a concentrated phosphoprotein fraction amenable to downstream LC-MS/MS, conventional SDS-PAGE, or targeted immunoblotting [88]. This enrichment step is particularly advantageous for plant or yeast extracts, where the abundance of storage or host proteins can obscure low-level phosphoproteins such as recombinant caseins.

Diagonal electrophoresis using Phos-tag™ gels provides an additional method for identifying phosphorylated proteins within complex samples. In this two-dimensional approach, proteins are first separated by conventional SDS-PAGE in the first dimension, followed by separation with Phos-tag SDS-PAGE in the second dimension. Non-phosphorylated proteins migrate along the diagonal, while proteins that were initially phosphorylated shift off the diagonal due to the loss of Phos-tag-mediated mobility retardation following dephosphorylation. This strategy facilitates the direct discrimination of phosphorylated isoforms without requiring prior enrichment and allows for the systematic identification of phosphorylation events in recombinant caseins expressed in plant or yeast systems. When combined with subsequent Western blotting using anti-casein-specific antibodies, diagonal electrophoresis provides an additional layer of resolution in phosphorylation mapping, complementing both single-dimension Phos-tag SDS-PAGE and mass-spectrometric analyses [89,90].

Despite their advantages, Phos-tag-based approaches have limitations when applied to recombinant caseins within complex cell lysates, where abundant host proteins and other phosphorylated species can obscure the detection of low-abundance casein phospho-isoforms. The high density and heterogeneity of phosphorylation sites in caseins often result in overlapping band patterns on Phos-tag gels, complicating interpretation. Furthermore, if recombinant caseins lack phosphorylation, Phos-tag gels yield a single band indistinguishable from dephosphorylated controls. Therefore, it is essential to confirm the phosphorylation status of recombinant caseins using mass spectrometry or kinase assays before employing Phos-tag strategies to ensure methodological suitability and efficient resource use.

Mass spectrometry remains the benchmark for precision and depth, delivering exact site assignments and phosphorylation stoichiometry. Gel-based methods (SDS-PAGE, Urea-PAGE, and Phos-tag SDS-PAGE) serve as rapid screens, require minimal instrumentation, and reveal gross phosphorylation differences, but they lack the sensitivity and site-specificity of MS. Phos-tag technology partially bridges this gap by offering isoform-resolved separation and selective enrichment, yet definitive site mapping still relies on tandem MS. These methods collectively provide a comprehensive approach to analyzing casein phosphorylation, not only revealing critical information about the functionality of recombinant proteins but also ensuring their safety and efficacy in various applications.

## 6. Enhancing Casein Phosphorylation in Heterologous Systems: Strategies and Considerations

**Figure 3:**
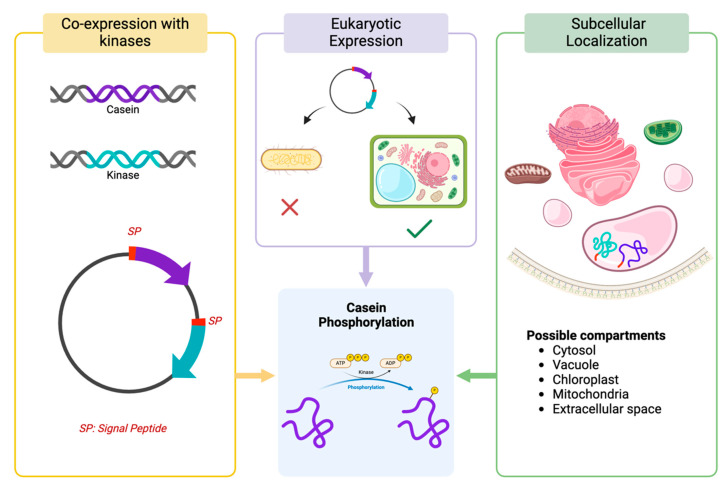
Proposed strategies to enhance casein phosphorylation in heterologous expression systems. Created in BioRender. Mora vasquez, S. (2025) https://BioRender.com/txbbxec (accessed on 24 June 2025).

As caseins expressed in heterologous systems, such as bacteria or plants, lack phosphorylation, several strategies can be explored to either mimic this modification or enable post-translational phosphorylation within these systems. These approaches include co-expression with kinases, utilizing eukaryotic expression systems, and employing signal peptides for targeted subcellular localization. A summary of these strategies is presented in Figure 3.

1 Co-expression with Kinases:

One of the primary challenges in achieving efficient phosphorylation of caseins in heterologous systems stems from the absence of native kinases that specifically target these proteins. Fam20C, a kinase predominantly active in the secretory pathway of mammalian cells, has been identified as the principal enzyme responsible for the phosphorylation of serine residues in caseins [91]. Its inclusion in heterologous systems, therefore, presents a solution to address the phosphorylation deficit observed during casein expression in non-mammalian systems. This approach has already led to multiple patents, underscoring its potential for industrial applications and reinforcing its feasibility as a viable strategy for casein phosphorylation.

The co-expression of casein proteins and Fam20C within the same system could potentially enable proper post-translational modification. This involves designing recombinant vectors to simultaneously express both the casein and Fam20C genes, with careful optimization of their expression levels to ensure sufficient amounts of Fam20C are available to phosphorylate the caseins. A mismatch in expression levels, such as overexpression of either component, could hinder efficient phosphorylation—either due to insufficient kinase or saturation of the kinase by excessive substrate. While still in the experimental phase, this strategy represents a promising avenue for addressing the challenges of phosphorylation in heterologous systems.

2 Using Eukaryotic Expression Systems:

While *E. coli* is frequently employed for recombinant protein production, its lack of an intrinsic secretory pathway makes it an unsuitable candidate for the production of caseins. Eukaryotic expression systems possess the natural machinery for post-translational modifications. Yeast and plant cell lines are more suitable for achieving phosphorylation, as they possess key cellular components, including kinases and the necessary subcellular machinery, to partially replicate the natural phosphorylation process. While not identical to mammalian systems, these eukaryotic hosts provide a closer approximation to the native environment required for post-translational modifications such as phosphorylation.

3 Use of Signal Peptides for Subcellular Localization

In plants, improper protein localization can significantly impair phosphorylation efficiency. This is primarily due to the presence of proteases and phosphatases, which are distributed across various compartments such as the cytosol, vacuole, chloroplast, mitochondria, and extracellular space. For instance, compartments like the vacuole or extracellular space may have proteases with high activity, which can degrade recombinant proteins, leading to reduced yield. Conversely, targeting proteins to compartments with limited protease activity, such as the endoplasmic reticulum (ER) or chloroplasts, may enhance protein stability and accumulation [92]. Thus, the use of signal peptides to direct both proteins (casein and kinase) to the same subcellular location is essential for enhancing phosphorylation outcomes.

## 7. Conclusions

Addressing the challenge of insufficient phosphorylation in heterologous systems requires a strategic approach tailored to the desired level of control, functionality, and precision. Potential solutions include co-expression with kinases, transitioning to eukaryotic expression systems, and optimizing subcellular localization to mimic native phosphorylation processes more effectively. These strategies, particularly when used in combination, could yield biologically accurate results that enhance the functionality and stability of recombinant casein proteins. Ultimately, the choice of approach will depend on the specific objectives of the research or industrial application and the extent to which phosphorylation contributes to achieving bioequivalence and native mimicry in the expressed proteins.

It is also important to note that while the current literature qualitatively demonstrates the role of phosphorylation in modulating casein calcium-binding and curd formation properties, systematic studies quantifying the functional thresholds (e.g., calcium-binding loss percentages or curd formation efficiency minima) associated with specific phosphorylation levels in recombinant caseins remain limited. Future investigations addressing these quantitative relationships will be crucial for establishing functional benchmarks that can guide the assessment and optimization of phosphorylation in recombinant casein production for research and industrial applications.

## Data Availability

No new data were created or analyzed in this study. Data sharing is not applicable to this article.

## Abbreviations

The following abbreviations are used in this manuscript: SDS-PAGE, sodium dodecyl sulfate-polyacrylamide gel electrophoresis; WB, Western blot; FPLC, fast protein liquid chromatography; Ni-NTA, nickel-nitrilotriacetic acid; HPLC, high-performance liquid chromatography; LC-ESI-MS, liquid chromatography-electrospray ionization mass spectrometry; MALDI-MS, matrix-assisted laser desorption/ionization mass spectrometry.
